# GLUT-1 Expression in Breast Cancer

**DOI:** 10.5146/tjpath.2021.01557

**Published:** 2022-05-19

**Authors:** Oguzhan Okcu, Bayram Sen, Cigdem Ozturk, Gulname Fındık Guvendi, Recep Bedir

**Affiliations:** Department of Pathology, Recep Tayyip Erdoğan University Training and Research Hospital, Rize, Turkey; Department of Biochemistry, Recep Tayyip Erdoğan University Training and Research Hospital, Rize, Turkey

**Keywords:** Breast cancer, Immunohistochemistry, GLUT-1

## Abstract

*
Objective:* Numerous studies have been conducted to predict the prognosis of breast cancers. The effect of glucose transporter protein 1 (GLUT-1), the main carrier protein responsible for glucose transport, was investigated in breast cancer patients.

*
Material and Method:* 170 patients operated for breast carcinoma were included in this study. We analysed the prognostic significance of GLUT-1 immune-expression in 149 patients without neoadjuvant therapy, and in 21 patients with neoadjuvant therapy.

*
Results:* GLUT-1 expression was correlated with poor prognostic factors such as estrogen receptor and progesterone receptor negativity, high Ki-67 proliferation index, and high histological and nuclear grade (p<0.001). GLUT-1 was expressed at a statistically higher rate in invasive ductal carcinomas, compared to invasive lobular carcinomas (p <0.001), and was expressed at a higher rate in luminal B, human epidermal growth factor receptor 2 and triple-negative molecular subtypes compared to luminal A subtype tumors (p <0.001). There was no statistically significant difference between GLUT-1 expression and presence of neoadjuvant therapy. Univariate survival analysis showed high GLUT1 expression was associated with low disease-free survival.

*
Conclusion:* GLUT-1 expression was found to be associated with poor pathological prognostic factors in breast carcinoma patients. The results suggest that GLUT-1 expression can be considered as a prognostic marker in breast cancers, and it may be used as a target molecule in personalized treatment approaches.

## INTRODUCTION

According to the Globocon 2018 data, breast cancers are the most common cancer in 11 regions worldwide and the first cause of cancer-related deaths in women. Around 2 million new patients and 600,000 deaths were recorded worldwide in 2018 (1).

The most important prognostic factors used in the treatment planning of patients are the TNM staging system and hormone receptor expression profiles (2). A very different prognosis is observed among patients of a similar stage. Therefore, different markers remain the subject of research to identify patients with poor prognosis and to develop new individualized treatment modalities.

Glucose transporter protein 1 (GLUT-1) is the main carrier protein responsible for physiological and pathological glucose transport. The expression of GLUT-1 increases with the effect of hypoxia and decreased oxidative phosphorylation to meet the increasing energy need of tumor cells for proliferation, invasion, and metastasis (3–5).

In our study, we aimed to determine the prognostic significance of GLUT-1 expression in breast cancer patients, the relationship between GLUT-1 expression level and clinicopathological prognostic parameters, and the effect of neoadjuvant therapy on GLUT-1 expression.

## MATERIALS and METHODS

### Study Design and Case Selection

Patients treated with invasive breast carcinoma with mastectomy +/- axillary lymph node materials operated in our center between January 2017 and April 2020 were retrospectively scanned from the departmental databases. Paraffin blocks, hematoxylin-eosin slides, and immunohistochemical slides (estrogen receptor (ER), progesterone receptor (PR), human epidermal growth factor receptor 2 (HER 2), Ki-67) were retrieved from the pathology archives. Patients whose materials could not be found, and whose clinical data could not be reached were excluded from the study. A total of 170 female patients were included in the study.

Mastectomy + axillary lymph node dissection was performed in 154 patients, and simple mastectomy in 16 patients. Neoadjuvant chemotherapy treatment was applied in 21 of the patients.

Two different study groups were formed according to neoadjuvant treatments. The prognostic significance of GLUT-1 expression and its relationship with clinicopathological parameters were evaluated in the main study group of 149 patients who did not receive neoadjuvant therapy. The effect of neoadjuvant therapy on GLUT-1 expression was evaluated in the second study group consisting of 21 patients who received neoadjuvant therapy.

Slides stained with Hematoxylin-eosin and with immu-nohistochemical markers for ER (SP1, Ventana), PR (1E2, Ventana), HER2 (4B5, Ventana), and Ki-67 (30-9, Ventana) were re-evaluated and re-scored by 2 pathologists (O.O., Ç.Ö.). For each case, a paraffin block containing sufficient tumor area was determined to apply GLUT-1 immunohistochemical antibody.

### Patient Data

Gender, age, survival time, development of metastasis, and data of recurrence were obtained from the hospital and national electronic databases. In addition, pathological data such as tumor diameter, pathological stage, nuclear grade, histological grade, axillary lymph node metastasis were obtained from the pathology reports.

### Outcomes

Disease-free survival is defined as the time to clinical, radiological, or pathological metastasis/recurrence after major surgery or the time to the last follow-up. Unfortunately, we could not analyze overall survival due to the short follow-up period.

### Histopathological and Immunohistochemical Staining

A 4 μm section from each formalin-fixed, paraffin-embedded tumor tissue block containing all morphological features of the tumor was selected for the study. Colon carcinoma tissue was added as the positive control, and benign breast parenchyma was designated as the negative control. The Ventana Medical System (SN: 714592, Ref: 750-700 Arizona, USA) automated immunohistochemistry device was used. Immunohistochemical staining was performed using UltraView Universal DAB Detection Kit (REF: 760-500, Ventana), and GLUT1 antibody (PA1-46152, 1/200 diluted, GLUT1 Rabbit Polyclonal Antibody).

The cytoplasmic and membranous staining pattern was accepted as positive staining for the GLUT-1 antibody. Specimens were scored according to the intensity of staining (0- no staining; 1- weak; 2- moderate; 3- strong), and the extent of tumor cells stained (<10% were scored as 0; 10-25% as 1 point; 26-50% as 2 points; and > 50% as 3 points).

**Figure 1:**
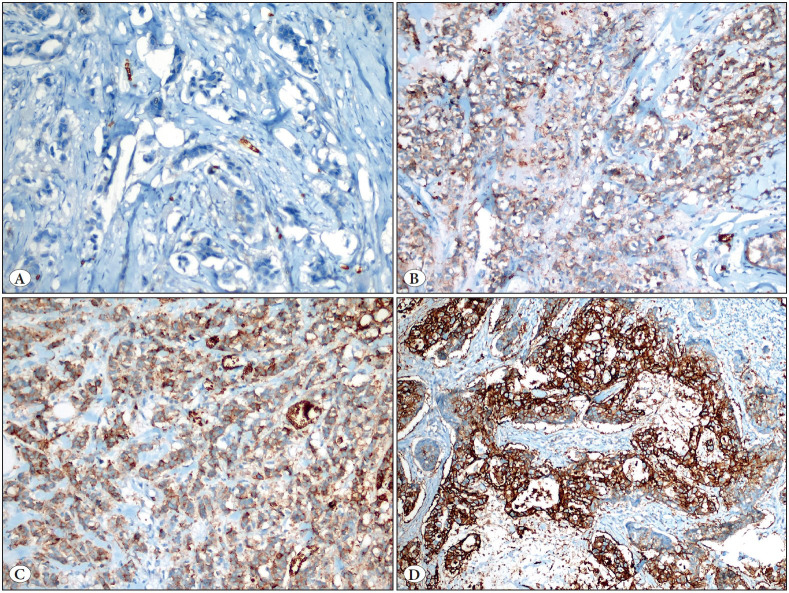
**A)** No staining with GLUT-1 antibody (GLUT x400), **B)** Weak intensity of GLUT-1 immunoreactivity (GLUT x200), **C)** Moderate intensity of GLUT-1 immunoreactivity (GLUT x200), **D)** Strong intensity of GLUT-1 immunoreactivity in tumor (GLUT x200).

After the evaluation, intensity and extensity scores were summed up for statistical analysis, and > 2 points were accepted as positive while ≤ 2 points were considered as negative GLUT-1 final scores (6) (Figure 1A-D).

Patients with a staining prevalence of more than 1% for ER and PR were accepted as positive. Patients with complete membranous staining in more than 10% tumor cells for HER 2 were accepted as score 3 (positive), weak to moderate complete membrane staining in more than 10% of tumor cells, or complete membrane staining in less than 10% of tumor cells were accepted as score 2. More than 10% incomplete weak membranous staining and no staining were considered negative (7,8). Since additional studies such as SISH and FISH could not be performed at our center, cases with a HER2 score of 2 were classified as HER2 status unknown. The Ki-67 proliferation index was evaluated in the area where the hot spot staining was observed. A Ki-67 proliferation index ≥14% were considered as high, and <14% were considered as low Ki-67 proliferation index (9).

The patients were divided into surrogate subtypes (luminal A (LA)(ER+, PR+/-, HER2 -, low Ki-67), luminal B (LB)( ER+, PR+/-, HER2+ or ER+, PR+/-, HER2-, high Ki-67) , HER2 (ER-,PR-, HER2+), and triple-negative (TN)( ER-, PR-, HER2-)) according to the ER, PR, HER2, and Ki-67 immunohistochemical marker staining patterns (10,11). All patients were divided into histological types according to the World Health Organization breast cancer classification (11), and graded according to the Nottingham histological grade scoring system (12).

### Statistical Analysis

Statistical analysis was performed using SPSS version 21 software. The compliance of numerical variables to a normal distribution was evaluated using the Kolmogorov-Smirnov test and histogram graphics. Continuous variables not conforming to a normal distribution were compared between groups using the Mann-Whitney U test. Whether there was a difference between the groups in terms of categorical variables was evaluated using the Chi-square (Pearson Chi-square, Linear-by-linear association) and Fisher’s Exact tests. Clinicopathological variables predicting disease-free survival was investigated by univariate analyzes using the Log-rank test and Cox regression analysis. Variables with p<0.2 as determined by univariate analyses were selected as covariates, and independent risk factors predicting disease-free survival were analyzed using the backward method Cox regression analysis. Survival rates were calculated by Kaplan-Meier survival analysis. For statistical significance, the p value was set as <0.05.

## RESULTS

### General Characteristics

The mean age of the 149 patients was 59.4 (range, 31-91). The tumor size was 0.5-14 cm, and the mean tumor diameter was 2.72 cm. 116 of the patients were invasive ductal carcinomas (IDC), 9 were invasive lobular carcinomas (ILC), 5 were mixed (ductal and lobular), and 19 were other types (tubular, mucinous, micropapillary, metaplastic carcinomas) of invasive breast carcinomas. The clinicopathological features of the patients are shown in Table 1.

**Table 1:** Association of glucose transporter protein 1 (GLUT-1) expression with clinicopathological parameters in 149 patients who did not receive neoadjuvant therapy.

**Variables**	**GLUT-1 **			
	** **	**Negative**	**Positive**	** **
	** **	**Count (%)**	**Count (%)**	p value*
Diameter	<2 cm	29 (41.4)	21 (26.6)	0.13
	2-5 cm	35 (50)	52 (65.8)	
	> 5 cm	6 (8.6)	6 (7.6)	
Molecular surrogate subtypes**	LA	48 (70.6)	17 (21.5)	<0.001
	LB	19 (27.9)	47 (59.5)	
	HER 2	1 (1.5)	7 (8.9)	
	TN	0 (0)	8 (10.1)	
Histologic type	IDC	46 (65.7)	70 (88.6)	0.005
	ILC	8 (11.4)	1 (1.3)	
	Mixed	3 (4.3)	2 (2.5)	
	Others	13 (18.6)	6 (7.6)	
Estrogen Receptor	Negative	1 (1.4)	16 (20.3)	<0.001
	Positive	69 (98.6)	63 (79.7)	
Progesterone Receptor	Negative	3 (4.3)	29 (36.7)	<0.001
	Positive	67 (95.7)	50 (63.3)	
Ki-67**	Low	53 (75.7)	16 (21.1)	<0.001
	High	17 (24.3)	60 (78.9)	
HER 2	Negative	59 (84.3))	54 (68.4)	0.004
	Positive	6 (8.6)	23 (29.1)	
	Unknown	5 (7.1)	2 (2.5)	
Nuclear Grade	1	9 (12.9)	1 (1.3)	<0.001
	2	58 (82.9)	58 (73.4)	
	3	3 (4.3)	20 (25.3)	
Histological Grade	1	12 (17.1)	1 (1.3)	<0.001
	2	55 (78.6)	59 (74.7)	
	3	3 (4.3)	19 (24.1)	
Angiolymphatic Invasion	No	40 (57.1)	42 (53.2)	0.626
	Yes	30 (42.9)	37 (46.8)	
Perineural Invasion	No	47 (67.1)	56 (70.9)	0.622
	Yes	23 (32.9)	23 (29.1)	
Multicentricity	No	54 (77.1)	70 (88.6)	0.062
	Yes	16 (22.9)	9 (11.4)	
Lymph node metastasis	No	36 (57.1)	35 (47.3)	0.25
	Yes	27 (42.9)	39 (52.7)	
Metastasis	No	68 (97.1)	69 (87.3)	0.035
	Yes	2 (2.9)	10 (12.7)	
Death	No	69 (98.6)	77 (97.5)	1.000
	Yes	1 (1.4)	2 (2.5)	

* P value below 0.05 is statistically significant ** We had missing data for some variables (3 patients for Ki-67 immunohistochemistry and 2 patients for molecular surrogate subtypes) *** **LA:** Luminal A, **LB:** Luminal B, **HER 2: **Human epidermal growth factor receptor 2, **IDC:** Invasive ductal carcinomas, **ILC:** Invasive lobular carcinoma

### GLUT-1 Expression and Clinicopathological Features

**Figure 2:**
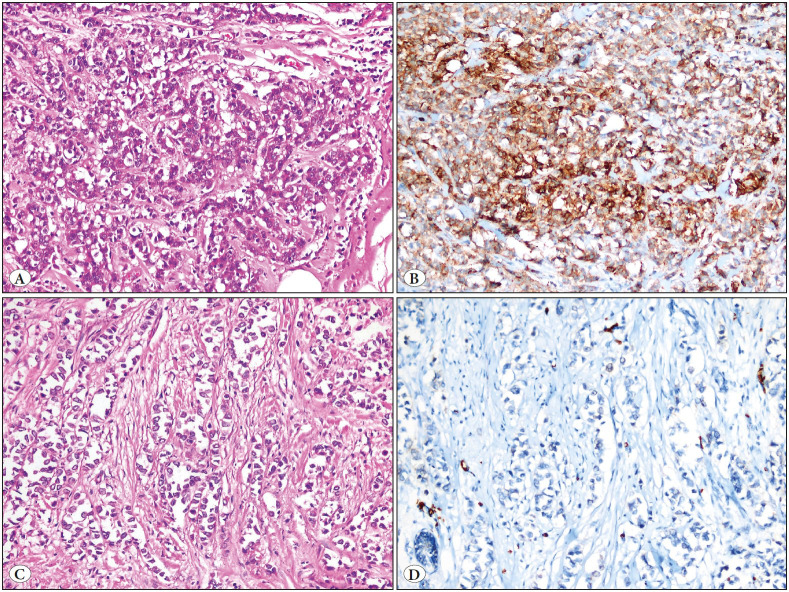
**A)** Invasive ductal carcinoma, intermediate nuclear grade (HE x200), **B)** Strong intensity of GLUT-1 immunoreactivity in invasive ductal carcinoma (GLUT x200), **C)** Invasive lobular carcinoma (HE x200), **D)** No staining with GLUT-1 in invasive lobular carcinoma (GLUT x200).

GLUT-1 expression was positive in 53% (79/149) of the patients. According to histological types and molecular surrogate subtypes, GLUT-1 was expressed at a statistically higher rate in IDC patients compared to ILC (p <0.001) (Figure 2A-D). GLUT1 was expressed at a higher rate in Luminal B, HER 2 and triple negative subtypes, compared to the Luminal A subtype cases (p <0.001).

GLUT-1 expression was positively correlated with poor prognostic factors such as ER and PR negativity, high Ki-67 proliferation index, and high histological and nuclear grade (p <0.001) (Table 1).

No statistically significant difference was found between GLUT-1 expression, and presence of neoadjuvant chemotherapy (Table 2).

**Table 2:** Glucose transporter protein 1 (GLUT-1) expression rates by neoadjuvant treatment status.

** **	** **	**GLUT-1**			
** **	** **	**Negative**	**Positive**	**Total**	p value*
Neoadjuvant chemotherapy	No, n (%)	70 (47)	79 (53)	149	0.112
	Yes, n (%)	6 (28.6)	15 (71.4)	21	

* P value below 0.05 is statistically significant

### GLUT-1 Expression and Survival Analysis

**Figure 3:**
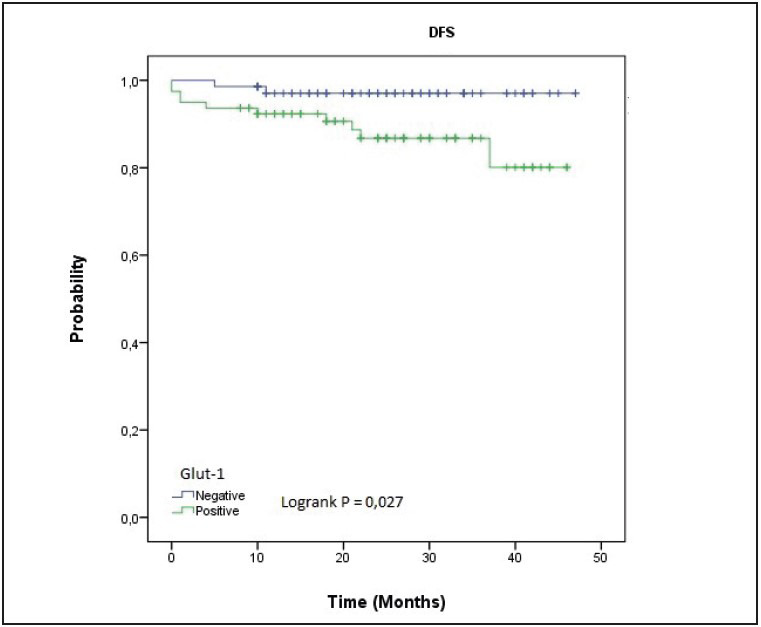
Kaplan-Meier disease free survival by GLUT-1 expression.

The follow-up periods of the patients varied between 8 to 47 months (median 26 months). In the survival analysis, disease-free survival (DFS) durations were found to be significantly shorter in patients with GLUT-1 expression compared to patients without GLUT-1 expression (40,65 months (95% CI: 37.57-43.79) vs. 45.86 months (95% CI 44.26-47.42) (log-rank p: 0.027) (Figure 3). Although DFS was shorter in patients with GLUT-1 positivity in univariate analysis; multivariant analysis revealed only size (>5 cm vs <2 cm) (HR: 31.376; 95% CI: 3.36- 292.968), and Ki-67 level (HR: 7.61; 95% CI: 1.478- 39.192) to predict low DFS (Table 3, Table 4).

**Table 3:** Univariate analysis results of clinicopathological variables associated with disease-free survival.

**Variables**	**B**	**SE**	**Wald**	**p**	**HR**	**95.0% CI for HR**	
						**Lower**	**Upper**
Age	0.017	0.022	0.636	0.425	1.018	0.975	1.062
GLUT-1	1.551	0.775	4.007	0.045	4.715	1.033	21.523
Estrogen Receptor	-1.526	0.615	6.152	0.013	0.217	0.065	0.726
Progesterone Receptor	1.425	0.578	6.072	0.014	4.159	1.339	12.923
Ki-67	1.658	0.776	4.564	0.033	5.251	1.147	24.043
Diameter ( > 5 cm vs. <2 cm)	3.109	1.122	7.684	0.006	22.399	2.486	201.814
HER 2	-0.993	1.045	0.904	0.342	0.37	0.048	2.87
Angiolymphatic Invasion	0.598	0.588	1.037	0.309	1.819	0.575	5.756
Perineural Invasion	0.935	0.578	2.615	0.106	2.547	0.82	7.91
Lymph Node Metastasis	0.68	0.63	1.165	0.28	1.974	0.574	6.792
Multicentricity	-0.76	1.045	0.53	0.467	0.467	0.06	3.625

* Cox regression analysis

**Table 4:** Independent risk factors associated with disease-free survival (Multivariate analysis).

** **	**B**	**SE**	**Wald**	**p**	**HR**	**95.0% CI for HR**	
						**Lower**	**Upper**
Diameter			15.772	0			
2-5 cm vs <2 cm	0.969	1.086	0.795	0.372	2.635	0.313	22.152
>5 cm vs <2 cm	3.446	1.14	9.14	0.003	31.376	3.36	292.968
Ki 67	2.029	0.836	5.889	0.015	7.61	1.478	39.192

* Cox regression model

## DISCUSSION

Glucose metabolism is one of the most significant steps in regulating cellular and systemic homeostasis and tumor carcinogenesis. Glucose transporter protein families provide glucose uptake from the systemic circulation into the cell (5,13,14). GLUT-1 is the main carrier protein found in many cells responsible for physiological and pathological glucose uptake. Their values in these cells change under physiological and pathological conditions. Especially the inhibition and activation of *RAS, SRC, c-MYC* and *P53* genes were associated with GLUT-1 expression levels (15,16). In non-neoplastic cells, the *p53* gene has been reported to inhibit GLUT-1 and GLUT-4, and the mutation in the *p53* gene has been reported to accelerate glucose transport for tumor cells by increasing the function of GLUT (17,18).

Various studies have shown that the use of GLUT-1 antibodies provides a reduction in tumor size as a result of apoptosis, and some molecules have an antiproliferative effect on tumor cells by causing GLUT-1 inhibition (19–21). This situation reveals the close connection between carcinogenesis and glucose metabolism of tumoral cells via glucose transporter proteins, and shows that glucose transporter proteins may be target molecules in cancer therapy. Targeting this pathway may lead to significant results in cancer treatment. In addition to these promising findings of GLUT-1 expression in cancer treatment, it has been also reported that GLUT-1 expression can be used diagnostically to differentiate benign and malignant urothelial tumors (22).

The prognostic significance of GLUT-1 expression in different cancer types such as osteosarcoma, gastric adenocarcinoma, esophagus adenocarcinoma, pancreatic carcinoma, lung carcinoma, oral squamous cell carcinoma, endometrial adenocarcinoma has been reported in numerous studies (23–30).

In the meta-analysis of Deng et al. (31), consisting of 1861 breast cancer patients, high GLUT-1 expression levels correlated with high histological grade, negative ER and PR, and low survival times. In addition, Krzeslak et al. (32) reported that GLUT-1 expression was observed in 50% of breast carcinoma patients and that GLUT-1 expression was detected at a higher rate in poorly differentiated tumors than well-differentiated tumors. Kang et al. (33) reported that GLUT-1 expression was associated with negative ER, PR, and high nuclear grade and poor prognosis in breast carcinoma patients. Hussein et al. (34) found higher GLUT-1 expression levels in IDC patients compared to ILC and mixed ductal and lobular carcinoma patients. In the same study, GLUT-1 expression was found to be associated with basal phenotype breast carcinoma with high histological grade, negative ER and PR, and high p53 expression level.

Similar to the studies reported in the literature, GLUT-1 expression was higher in Luminal B, HER2 and, triple negative subtypes compared to Luminal A subtype cases in our study. Additionally, GLUT-1 expression was found to be statistically correlated with poor prognostic parameters such as high histological and nuclear grade, negative ER and PR expression, and high Ki-67 proliferation index. In the survival analysis, disease-free survival (DFS) durations were found to be significantly shorter in patients with GLUT-1 expression. GLUT-1 expression was not associated with tumor size, axillary lymph node metastasis, angiolymphatic invasion, perineural invasion, and multicentricity variables.

These findings suggest that the evaluation of GLUT-1 expression in breast cancers may be a promising parameter. However, studies with larger cohorts and longer follow-up periods are necessary in order to bring GLUT1 expression analysis to daily practice.

In our study, GLUT-1 expression was found to be statistically significantly lower in ILC patients compared to IDC patients. The reasons for this result might be the energy metabolism mediated by a different glucose transporter protein in the development of ILC or low GLUT-1 levels that cannot be evaluated immunohistochemically in ILC cases.

To our knowledge, there is no study investigating the relationship between GLUT-1 expression and neoadjuvant therapy in breast cancer patients. In our study, GLUT-1 expression was compared in two groups; patients with or without neoadjuvant therapy. Although GLUT-1 expression was higher in patients receiving neoadjuvant therapy (71.4% vs. 54%), no significant difference was observed between these two groups. The reason might be that current neoadjuvant therapy applications have no affect over GLUT-1, or the small number of patients with neoadjuvant therapy in our study.

However, there are limitations in our study in this regard. In order to determine the relationship between GLUT-1 expression and neoadjuvant therapy, the comparison of GLUT-1 expression between the pretreatment core biopsy and post-treatment surgical materials may be more decisive. This was not in the design of our study.

## CONCLUSION

In our study, GLUT-1 expression was associated with pathological poor prognostic factors such as high histological and nuclear grade, ER and PR negativity, and low disease-free survival in breast carcinoma patients. These results suggest that GLUT-1 expression can be considered as a prognostic marker in breast cancers. It can be a promising target molecule for personalized treatment approaches. However, for the GLUT-1 molecule to be used in daily practice as a prognostic marker, our results should be supported by studies with longer follow-up periods and larger cohorts.

## Conflict of Interest

Authors declare no conflict of interest.
